# Special education teachers' involvement and perceived barriers to delivering oral health education for students with disabilities—A cross‐sectional study

**DOI:** 10.1111/ipd.13258

**Published:** 2024-08-06

**Authors:** Faris Yahya I. Asiri, Marc Tennant, Estie Kruger

**Affiliations:** ^1^ Department of Preventive Dental Sciences, College of Dentistry King Faisal University Al‐Ahsa Saudi Arabia; ^2^ International Research Collaboration—Oral Health and Equity, School of Allied Health The University of Western Australia Perth Western Australia Australia

**Keywords:** barriers, involvement, oral health education, oral health promotion, special education teacher, students with disabilities

## Abstract

**Background:**

Dental caries is prevalent among children, including those with disabilities. Although the World Health Organization recommends school‐based oral health promotion (OHP) programmes involving teachers, limited research has explored teachers' roles and perspectives.

**Aim:**

To assess special education teachers' involvement and difficulties regarding oral health education (OHE), attitudes towards OHP and barriers to oral healthcare access for students with disabilities (SWDs).

**Design:**

This descriptive cross‐sectional study, conducted in Al‐Ahsa, Saudi Arabia, involved 264 special education teachers using a validated, self‐administered questionnaire, and descriptive and analytical statistics were used for data analysis.

**Results:**

Only 39% of teachers incorporated OHE into their teaching, and just 20.8% received training for OHE delivery. Teachers showed strong support for integrating OHE into the curriculum (84.1%) and a no‐sugar policy (78%). There was, however, less support for school‐based toothbrushing (39%). OHE barriers included insufficient resources (56.1%), limited knowledge (29.2%) and misconceptions about primary teeth removal (47.4%). The three most common barriers to oral healthcare access were extended waiting lists (75.0%), long waiting times (73.1%) and fear of dental equipment (67.4%).

**Conclusion:**

This study highlights the need for collaboration between healthcare professionals, educators and parents to enhance OHE and reinforce OHP for SWDs within special education and beyond.

**Why this paper is important to paediatric dentists**

- Collaborative engagement between teachers, caregivers and dental professionals through school‐based OHP may be key to improving SWDs' oral health.
- Special education teachers may become more involved in OHE and OHP with proper information and resources and a supportive awareness programme highlighting the importance of primary teeth and the benefits of toothbrushing.
- A validated tool for assessing educators' roles and challenges in incorporating school‐based OHP enables consistent evaluation and ensures effectiveness.

## 1 INTRODUCTION

Oral health is fundamental to well‐being, encompassing the mouth, teeth and related facial structure health. Untreated dental caries affects 2.5 billion adults and 514 million children globally, leading to tooth infections, pain, tooth loss, nutritional issues, emotional stress, missed school and financial burdens.^1^

To enhance children's and adolescents' oral health, the World Health Organization recommends implementing school‐based oral health promotion (OHP) and involving teachers in planning, developing and reviewing oral health policies; training in oral health and disease prevention; monitoring health‐related issues; collaborating with school health services; advocating for preventive oral health care; engaging with the community regarding dental screenings; providing parental education; and role modelling. Teachers play a crucial role in this framework especially for children without access to dental clinics by reaching children during their early health development stages and providing essential health information.^2^ Early OHP becomes even more critical for young people with disabilities and special education needs, who typically have poorer oral health and more unmet dental needs than their peers, thus helping to reduce the need for invasive treatments and prevent health complications.^3^

In Saudi Arabia, individuals with disabilities depend heavily on caregivers for oral health maintenance, facing poor oral hygiene status, limited access to dental care and a general lack of awareness about oral health.^4^ This scenario underscores the significance of teachers' involvement in daily interactions with students and their families, which can significantly influence OHP.

Prior studies have emphasised the value and benefits of specialised health instruction for students with disabilities (SWDs) and their oral health outcomes.^5^, ^6^, ^7^ Little research, however, has focussed on the contributions and viewpoints of educators in this process. We address this by evaluating schoolteachers' engagement in oral health education (OHE), identifying possible obstacles and investigating attitudes towards incorporating OHE into curricula and the broader school environment, including initiatives such as at‐school toothbrushing and sugar‐free policies, and addressing barriers faced by caregivers concerning dental check‐ups for SWDs.

## 2 MATERIALS AND METHODS

### 2.1 Study design

This descriptive cross‐sectional study investigated teacher involvement, perceived barriers to OHE and attitudes towards OHP in government schools within Saudi Arabia's Al‐Ahsa governorate. It specifically targeted schools offering special education programmes for SWDs.

### 2.2 Study setting

We sampled special education teachers in schools with special education programmes across Saudi Arabia's Al‐Ahsa governorate, within the Eastern Province.

### 2.3 Sampling

Using a decentralised, multistage sampling technique, we engaged various schools in the Al‐Ahsa governorate with special education programmes. Invitations were sent to the Department of Special Education and school principals, facilitated by the Planning and Development Department. Each school independently identified eligible teachers, ensuring at least 6 months' experience teaching SWDs for at least 10 h per week. Consecutive sampling was then employed to select eligible teachers sequentially as they became available.

### 2.4 Sample size

We used an online calculator (Raosoft, http://www.raosoft.com/samplesize.html) to determine an appropriate sample size based on an absolute precision criterion of 5% and a 95% confidence level. We determined the absolute precision parameter based on the expected SWD teacher population in the Al‐Ahsa governorate (i.e., 820) according to the latest data from Saudi Arabia's Ministry of Education.^8^ We input the absolute precision parameter and expected teacher population into the online sample size calculator, and the minimum sample size was 262.

### 2.5 Instrumentation

A questionnaire was developed for this study by selecting a previously published survey that aligned with the general objectives of our research.^9^ To ensure its relevance to our study context and research goals, we expanded the questionnaire by adding items carefully crafted to capture our variables of interest. We reviewed the existing literature, sought input from experts and conducted pilot tests. Through this systematic approach, new items were tailored to address essential OHP elements and barriers. We then validated the survey questionnaire containing the previously validated items and the newly added ones. We assessed face validity by piloting the questionnaire with five special education teachers. They determined whether items should be included and provided feedback on the wording's clarity. They were excluded from the study to avoid bias from their prior exposure to the questionnaire, which could influence their responses or perceptions. The questionnaire was divided into five sections: teachers' demographic characteristics, teachers' OHE involvement, perceived barriers to OHE in school settings, attitudes towards integrating OHP and OHE into school curricula and perceived barriers regarding SWDs' access to dental health care.

### 2.6 Data collection

Data collection occurred from February to June 2023. The invitation letter and the link to the questionnaire were sent to the Planning and Development Department of the General Administration of Education in Al‐Ahsa, who then sent invitation emails to the Department of Special Education and school principals, which were forwarded to special education teachers. Each school independently identified eligible teachers and forwarded the survey via school email addresses. The emails contained comprehensive study details, invitations to participate and links to the anonymous online questionnaire. Before completing the questionnaire, participants provided informed consent online. Ethics approval for the study was obtained from the Human Research Ethics Committee at the University of Western Australia (file reference 2022/ET000328).

### 2.7 Data analysis

We analysed the data using SPSS version 24, calculating means and standard deviations for continuous variables, and frequencies and percentages for categorical variables. Missing values were excluded from the analysis for data integrity. The questionnaire's internal consistency and reliability were checked using a Cronbach's alpha. The alpha values for teachers' perceptions of barriers to OHE and accessing oral health care were 0.73 and 0.60, respectively, indicating moderate‐to‐strong internal consistency. We used the Kolmogorov–Smirnov test and normal Q–Q plots to examine the normality of all continuous data, and we employed Levene's test to examine the homogeneity of variance. Because the assumptions of normality and equal variance were violated, we conducted comparisons using the nonparametric Mann–Whitney *U* and Kruskal–Wallis tests.

To categorise teachers' OHE involvement (nine questions, total score 9), correct answers were scored as one; incorrect responses were scored as zero. Teachers' involvement was divided into three categories using a 25th–75th‐percentile threshold: poor (25th percentile), medium (25th–75th percentile) and good (>75th percentile).

Perceived OHE barriers were evaluated using a 5‐point Likert scale (strongly disagree to strongly agree), aggregated into a 3‐point Likert scale (disagree, neutral and agree) for analysis. We used Pearson's chi‐squared test to determine whether participants' involvement in OHE was associated with demographics or other factors. We employed Fisher's exact test if the Pearson's chi‐squared test assumption was not satisfied. *p* < .05 was considered statistically significant.

## 3 RESULTS

**FIGURE 1:**
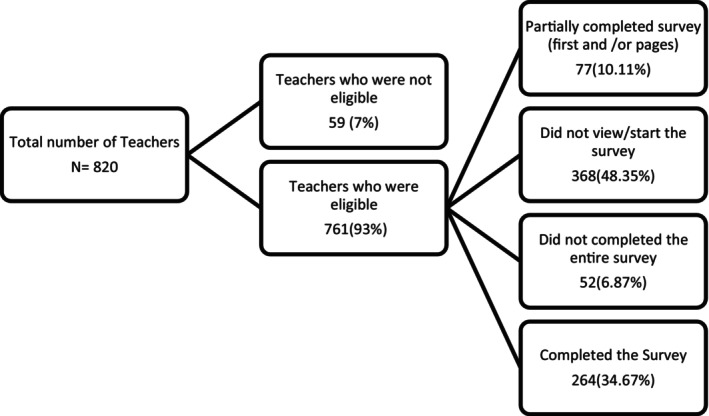
Flow chart of participant recruitment.

A total of 264 special education teachers were recruited from various educational settings for SWDs in Al‐Ahsa. The survey achieved a response rate of 34.65%, with 264 teachers fully completing the survey of 761 who were eligible. This is slightly above the mean reported in similar, previously published articles.^10^, ^11^ Figure 1 shows a detailed participant flow chart. The participants' ages ranged from 23 to 60 years; the average age was 37.93 ± 7.39, and most fell into the age categories of 23–35 years (49.6%) and 36–40 years (28.3%). Nearly equal percentages of male (51.5%) and female (48.4%) teachers participated, and 81.4% had bachelor's degrees. Over half the respondents (54.2%) indicated that their teaching included one‐to‐one and group instruction. In total, 61.7% had more than 8 years' experience working with SWDs; 43.2% worked in schools that provided inclusive classrooms for SWDs (Table 1).

**TABLE 1:** Demographic characteristics of special education teachers.

Characteristics	*n*	%
Gender		
Male	136	51.5
Female	128	48.5
Age		
23–35	131	49.6
36–48	101	38.3
49–60	32	12.1
Qualification		
University graduate	215	81.4
Masters/Higher diploma	47	17.8
PhD	2	0.8
Number of years in special education teaching		
1–5	40	15.2
6–8	61	23.1
>8	163	61.7
Mode of teaching		
Individual	87	33.0
Group	34	12.9
Both	143	54.2
Type of school and activity		
Specialised school/Institute	48	18.2
Self‐contained classroom	102	38.6
Inclusive classroom	114	43.2
Assisted by a teaching assistant		
Yes	30	11.4
No	234	88.6

Table 2 shows teachers' OHE involvement. Overall, 39% of teachers reported implementing OHE in curricula, but just 20.8% received OHE training from dental professionals. Rewarding students with sugary foods was reported by 25.4% of teachers. Additionally, 42.2% of teachers taught students about toothbrushing techniques, and 39.8% educated them about dentists. Only 32.6% of teachers, however, reported that caregivers desire OHE in schools. Furthermore, just 18.9% of teachers provided instruction to caregivers on toothbrushing. Additionally, 24.2% of teachers reported asking caregivers about students' dental check‐ups, and 25% reported educating them on the significance of maintaining children's dental health.

**TABLE 2:** Comparison of special education teachers' involvement in oral health education based on age, gender and experience teaching students with disabilities.

Questions												
Response	*n* (%)	Age			*p*	Gender		*p*	Teaching experience in years			*p*
		25–35	36–48	49–60		Male	Female		6 months–5 years	6–8	>8	
Curriculum includes oral health education												
Yes	103 (39)	58 (44.3)	35 (34.7)	10 (31.3)	.340	49 (36.0)	54 (42.2)	.470	13 (32.5)	27 (44.3)	63 (38.7)	.512
No	140 (53)	65 (49.6)	55 (54.5)	20 (62.5)		74 (54.4)	66 (51.6)		23 (57.5)	32 (52.5)	85 (52.1)	
Don't know	21 (8)	8 (6.1)	11 (10.9)	2 (6.3)		13 (9.6)	8 (6.3)		4 (10.0)	2 (3.3)	15 (9.2)	
Oral health education desired by caregivers												
Yes	86 (32.6)	45 (34.4)	33 (32.7)	8 (25.0)	.470	45 (33.1)	41 (32.0)	.334	9 (22.5)	24 (39.3)	53 (32.5)	.181
No	120 (45.5)	53 (40.5)	50 (19.5)	17 (53.1)		66 (48.5)	54 (42.2)		17 (42.5)	25 (41.0)	78 (47.9)	
Don't know	58 (22.0)	33 (25.2)	18 (17.8)	7 (21.9)		25 (18.4)	33 (25.8)		14 (35.0)	12 (19.7)	32 (19.6)	
Trained by dental professionals to provide oral health education												
Yes	55 (20.8)	23 (17.6)	25 (24.8)	7 (21.9)	.082	30 (22.1)	25 (19.5)	.823	5 (12.5)	11 (18.0)	39 (23.9)	.003*
No	156 (59.1)	74 (56.5)	59 (58.4)	23 (71.9)		83 (61.0)	73 (57.0)		18 (45.0)	38 (62.3)	100 (61.3)	
Don't know	53 (20.1)	34 (26.0)	17 (16.8)	2 (6.3)		29 (16.9)	30 (23.4)		17 (42.5)	12 (19.7)	24 (14.7)	
Caregivers taught how to brush their children's teeth												
Yes	50 (18.9)	26 (19.8)	18 (17.8)	6 (18.8)	.481	24 (17.6)	26 (20.3)	.759	6 (15.0)	10 (16.4)	34 (20.9)	.801
No	184 (69.7)	93 (71.0)	67 (66.3)	24 (75.0)		95 (69.9)	89 (69.5)		30 (75.0)	45 (73.8)	109 (66.9)	
Don't know	30 (11.4)	12 (9.2)	16 (15.8)	2 (6.3)		17 (12.5)	13 (10.2)		4 (10.0)	6 (9.8)	20 (12.3)	
Students taught about toothbrushing techniques												
Yes	112 (42.4)	47 (35.9)	50 (49.5)	15 (46.9)	.259	53 (39.0)	59 (46.1)	.382	11 (27.5)	31 (50.8)	70 (42.9)	.198
No	103 (39.0)	57 (43.5)	33 (32.7)	13 (40.6)		54 (39.7)	49 (38.3)		18 (45.0)	21 (34.4)	64 (39.3)	
Don't know	49 (18.6)	27 (20.6)	18 (17.8)	13 (40.6)		29 (21.3)	20 (15.6)		11 (27.5)	9 (14.8)	29 (17.8)	
Students taught about dentists												
Yes	105 (39.8)	51 (38.9)	41 (40.6)	13 (40.6)	.985	54 (39.7)	51 (39.8)	1.000	15 (37.5)	21 (34.4)	69 (42.3)	.567
No	126 (47.7)	63 (48.1)	47 (46.5)	16 (50.0)		65 (47.8)	61 (47.7)		21 (52.5)	29 (47.5)	76 (46.6)	
Don't know	33 (12.5)	17 (13.0)	13 (12.9)	3 (9.4)		17 (12.5)	16 (12.5)		4 (10.0)	11 (18.0)	18 (11.0)	
Students rewarded with sugary food												
Yes	67 (25.4)	32 (24.4)	20 (19.8)	15 (46.9)	.029*	33 (24.3)	34 (26.6)	.904	15 (37.5)	9 (14.8)	43 (26.4)	.132
No	183 (69.3)	92 (70.2)	74 (73.3)	17 (53.1)		96 (70.6)	87 (68.0)		23 (57.5)	49 (80.3)	111 (68.1)	
Don't know	14 (5.3)	7 (5.3)	7 (6.9)	0 (0.0)		7 (5.1)	7 (5.5)		2 (5.0)	3 (4.9)	9 (5.5)	
Caregivers asked about their children's regular dental visits												
Yes	64 (24.2)	28 (21.4)	26 (25.7)	10 (31.3)	.602	35 (25.7)	29 (22.7)	.270	9 (22.0)	14 (23.0)	41 (25.2)	.115
No	161 (61.0)	80 (61.1)	62 (61.4)	19 (59.4)		77 (56.6)	84 (65.6)		24 (60.0)	32 (52.5)	105 (64.4)	
Don't know	39 (14.8)	23 (17.6)	13 (12.9)	3 (9.4)		24 (17.6)	15 (11.7)		7 (17.5)	15 (24.6)	17 (10.4)	
Caregivers taught the importance of maintaining their children's dental health												
Yes	66 (25.0)	35 (26.7)	23 (22.8)	8 (25.0)	.346	35 (25.7)	31 (24.2)	.848	11 (27.5)	18 (29.5)	37 (22.7)	.048*
No	170 (64.4)	78 (59.5)	69 (68.3)	23 (71.9)		88 (64.7)	82 (64.1)		55 (55.0)	33 (54.1)	115 (70.6)	
Don't know	28 (10.6)	18 (13.7)	9 (8.9)	1 (3.1)		13 (9.6)	15 (11.7)		7 (17.5)	10 (16.4)	11 (6.7)	

*Note*: None of the expected frequencies were less than 20% of the total observations.

* Pearson's chi‐squared test significant at *p* < .05.

Teachers' involvement was further analysed to examine associations between age, teaching experience and gender. Age had no statistically significant associations with teachers' OHE involvement. Similarly, no statistically significant association was found between gender and OHE involvement. A statistically significant difference, however, was observed regarding age and rewarding students with sugary food (*p* = .029). A higher proportion of teachers over 35 did this (*p* = .029).

A statistically significant association was observed between teaching experience and receiving OHE training from dental professionals (*p* = .003). Teachers with over 8 years' experience showed the highest percentage of teaching caregivers about maintaining children's dental health. This difference was statistically significant compared to teachers with fewer than 8 years' experience (*p* = .048).

Table 3 shows teachers' perceptions of OHE barriers. Environmental barriers were cited as the most significant, with 62.5% of teachers facing difficulties recommending or finding dentists who treat SWDs. Individual barriers included insufficient teaching resources (56.1%) and inadequate understanding regarding removing decayed (47.4%) and painful (45.8%) primary teeth. Additionally, 29.2% of teachers reported having insufficient OHE information. These findings underscore the significant individual barriers teachers encounter when engaging in OHE. Furthermore, when teachers were asked about obstacles with caregivers regarding OHE involvement, 38.7% stated that caregivers do not understand the importance of maintaining good dental hygiene. 42.8% of teachers, however, disagreed that caregivers are unwilling to learn about OHE. Students' lack of OHE information (56.4%) was also perceived as a barrier. Nevertheless, when asked whether SWDs are not receptive towards OHE, only 18.2% of teachers agreed. Moreover, there was a statistically significant difference in teachers' perceptions of barriers to implementing OHE between schools practising partial inclusion, where SWDs study alongside regular students, and full inclusion, where SWDs are fully integrated into regular classrooms, among the three groups of schools based on their activities related to inclusive practices (Kruskal–Wallis test, *H* (2) = 7.64, *p* = .022). Teachers' perceptions of OHE barriers, however, were not statistically significantly associated with other demographic characteristics.

**TABLE 3:** Special education teachers' perceived barriers to oral health education for students with disabilities.

Barriers	Strongly disagree, *n* (%)	Disagree, *n* (%)	Neutral, *n* (%)	Agree, *n* (%)	Strongly agree, *n* (%)
Individual					
*May give improper advice regarding*					
Removal of painful primary teeth	28 (10.6)	35 (13.3)	76 (28.8)	105 (39.8)	20 (7.6)
Removal of decayed primary teeth	27 (10.2)	55 (20.8)	61 (23.1)	99 (37.5)	22 (8.3)
Regular dental check‐ups are not paramount for children	140 (53.0)	99 (37.5)	13 (4.9)	8 (3.0)	4 (1.5)
*Insufficient information about oral health education*	11 (4.2)	81 (30.7)	95 (36.0)	71 (26.9)	6 (2.3)
*Insufficient teaching materials*	25 (9.5)	52 (19.7)	39 (14.8)	99 (37.5)	49 (18.6)
Caregivers					
Caregivers unwilling to learn oral health education	36 (13.6)	77 (29.2)	66 (25.0)	67 (25.4)	18 (6.8)
Caregivers do not understand the importance of maintaining oral hygiene	28 (10.6)	45 (17.0)	89 (33.7)	72 (27.3)	30 (11.4)
Environment					
Difficulty recommending/Finding a dentist who can treat students with disabilities	17 (6.4)	30 (11.4)	52 (19.7)	98 (37.1)	67 (25.4)
Students					
Insufficient information about oral health education	18 (6.8)	37 (14.0)	60 (22.7)	79 (29.9)	70 (26.5)
Children with disabilities are not receptive to the teaching of oral health education	30 (11.4)	121 (45.8)	65 (24.6)	39 (14.8)	9 (3.4)

**FIGURE 2:**
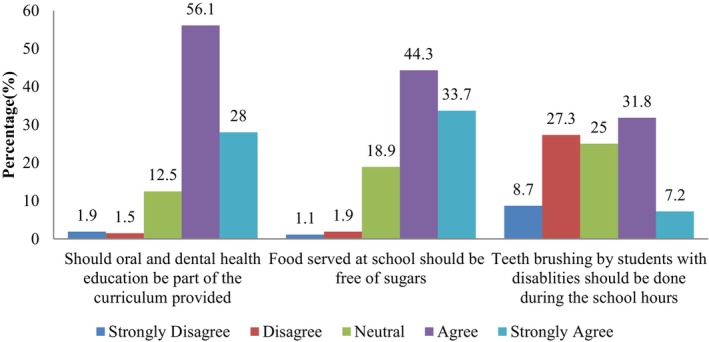
Integration of oral health promotion into the school curriculum.

Figure 2 shows special education teachers' attitudes towards school OHP. Most teachers (84.1%) displayed positive attitudes towards school OHP. They, however, reported challenges, such as facilitating toothbrushing by SWDs during school hours, which was only supported by 39.0% of teachers. By contrast, 78% supported no‐sugar policies. Similarly, 84.1% supported incorporating OHE into SWDs' curricula.

Table 4 shows perceived OHE barriers categorised by teachers' involvement (poor, medium and good). The responses indicate that the number of teachers within each involvement category who disagreed was neutral towards or agreed with the perceived OHE barriers. The *p*‐value of .004 indicates a statistically significant association between teachers' involvement and perceptions of providing improper advice regarding the removal of painful primary teeth. Among teachers with poor involvement, 53.5% agreed that they might give improper advice. This percentage decreased to 49.7% for medium involvement and 37.1% for good involvement. This suggests that higher OHE involvement correlates with a lower perception of providing improper advice. Similarly, teachers' perceptions of insufficient information about OHE showed a statistically significant association with teachers' involvement (*p* = .001). Specifically, among teachers with poor involvement, 44.2% agreed that there was insufficient OHE information, which decreased to 29.6% for medium involvement and 17.7% for good involvement. This suggests that as teachers' involvement in OHE increases, the perception of insufficient information decreases. No other barriers showed a statistically significant association with OHE involvement. Question about SWD toothbrushing during school hours showed a statistically significant association with teachers' involvement (*p* = .001). Specifically, among teachers with poor involvement, 39.5% disagreed with the idea, which decreased to 37.7% for medium involvement and to 29% for good involvement. This indicates that as teachers' involvement in OHE increases, support for toothbrushing at school by SWDs also increases.

**TABLE 4:** Comparison of special education teachers' involvement in oral health education, teacher‐perceived barriers to oral health education and oral health promotion integration into the school curriculum for students with disabilities.

Perceived barriers to oral health education	Teachers' involvement				
	Responses	Poor, *n* (%)	Medium, *n* (%)	Good, *n* (%)	*p*
Individual					
*May give improper advice regarding*					
Removal of painful primary teeth	Disagree	8 (18.6)	29 (18.2)	26 (41.9)	.004*
	Neutral	12 (27.9)	51 (32.1)	13 (21.0)	
	Agree	23 (53.5)	79 (49.7)	23 (37.1)	
Removal of decayed primary teeth	Disagree	14 (32.6)	42 (26.4)	26 (41.9)	.114
	Neutral	8 (18.6)	44 (27.7)	9 (14.5)	
	Agree	21 (48.8)	73 (45.9)	27 (43.5)	
Regular dental check‐ups are not paramount for children	Disagree	40 (93.0)	145 (91.2)	54 (87.1)	.176^a^
	Neutral	1 (2.3)	10 (6.3)	2 (3.2)	
	Agree	2 (4.7)	4 (2.5)	6 (9.7)	
Insufficient information about oral health education	Disagree	11 (25.6)	47 (29.6)	11 (17.7)	.001*
	Neutral	13 (30.2)	65 (40.9)	17 (27.4)	
	Agree	19 (44.2)	47 (29.6)	11 (17.7)	
Insufficient teaching materials	Disagree	14 (32.6)	41 (25.8)	22 (35.5)	.664
	Neutral	6 (14.0)	24 (15.1)	9 (14.5)	
	Agree	23 (55.5)	94 (59.1)	31 (50.0)	
Caregivers					
Caregivers unwilling to learn oral health education	Disagree	18 (41.9)	64 (40.3)	31 (50.0)	.057
	Neutral	17 (39.5)	38 (23.9)	11 (17.7)	
	Agree	8 (18.6)	57 (35.8)	20 (32.3)	
Caregivers not understanding the importance of maintaining oral hygiene	Disagree	13 (30.2)	41 (45.8)	19 (30.6)	.472
	Neutral	13 (30.2)	51 (32.1)	25 (40.3)	
	Agree	17 (39.5)	67 (42.1)	18 (29.0)	
Environment					
Difficulty recommending/finding a dentist who can treat students with disabilities	Disagree	10 (23.3%)	25 (15.7%)	12 (19.4)	.526
	Neutral	5 (11.6%)	33 (20.8%)	14 (22.6)	
	Agree	28 (65.1%)	101 (63.5%)	36 (58.1)	
Students					
Insufficient information about oral health education	Disagree	10 (23.3)	25 (15.7%)	12 (19.4)	.751
	Neutral	5 (11.6)	33 (20.8%)	14 (22.6)	
	Agree	28 (65.1)	101 (63.5%)	36 (58.1)	
Children with disabilities are unreceptive to the teaching of oral health education	Disagree	21 (48.8)	97 (61.0%)	33 (53.2)	.458
	Neutral	12 (27.9)	34 (21.4%)	19 (30.6)	
	Agree	10 (23.3)	28 (17.6%)	10 (16.1)	
Integration of oral health promotion into school environment					
Oral and dental health education should be part of the curriculum	Disagree	1 (2.3%)	4 (2.5%)	4 (6.5)	.457^a^
	Neutral	4 (9.3%)	19 (11.9%)	10 (16.1)	
	Agree	38 (88.4%)	136 (85.5%)	48 (77.4)	
Food served at school should be free of sugar	Disagree	3 (7.0%)	3 (1.9%)	2 (3.2)	.202^a^
	Neutral	6 (14.0%)	28 (17.6%)	16 (25.8)	
	Agree	34 (79.1%)	126 (80.5%)	44 (71.0)	
Toothbrushing should be done during school hours by students with disabilities	Disagree	17 (39.5)	60 (37.7)	18 (29.0)	.010*
	Neutral	15 (34.9)	42 (26.4)	9 (14.5)	
	Agree	11 (25.6)	57 (35.8)	35 (56.5)	

* Pearson's chi‐squared test significant at *p* < .05.

^a^ Fisher's exact test applied when assumptions were violated.

**FIGURE 3:**
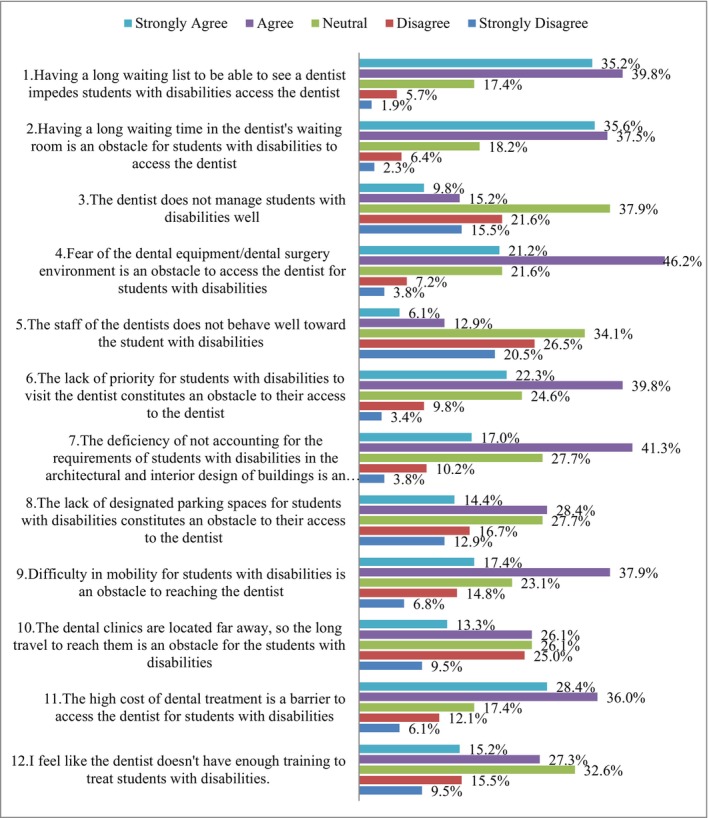
Teachers' perceptions of barriers to accessing oral healthcare services.

Figure 3 shows perceived barriers to accessing oral healthcare services. Most teachers felt that long waiting lists (75.0%) and times (73.1%) limited SWDs' access to dentists. Similarly, many believed that SWDs might find it difficult to maintain oral health due to their low prioritisation of dental visits (62.1%) and their fear of dental equipment (67.4%). Teachers agreed that a lack of appropriate facilities and inadequate carparking to meet SWDs' needs constituted barriers to oral healthcare access. Additionally, teachers reported SWDs' mobility problems (55.3%) and dental care's high costs (64.4%) as barriers. Moreover, 32.6% of teachers were unsure whether dentists had sufficient training to treat SWDs.

## 4 DISCUSSION

This comprehensive evaluation of special education teachers' involvement in delivering OHE to SWDs identifies factors related to integrating OHP in schools and special education teachers' perceived barriers to OHE and oral healthcare access in Al‐Ahsa. To the best of our knowledge, this is the first study of its kind. Gender had no statistically significant impact on the study variables; nevertheless, gender can affect regular teachers' knowledge and attitudes.^12^ This study revealed that only 39% of teachers included OHE in their curricula, aligning with articles indicating low levels of OHE in various parts of the world.^9^, ^13^, ^14^

This study revealed that most special education teachers lack formal training from dental professionals, which corroborates a cross‐sectional study in North Carolina, USA, where most teachers lacked training in incorporating oral health into early Head Start activities.^15^ Similarly, a quantitative study in KwaZulu‐Natal, South Africa, involving 154 regular schools found that 95% of teachers received no training.^14^ Furthermore, a study of special education teachers instructing children with autism in Singapore showed that most participants had not been taught to provide OHE by dental professionals.^9^ This underscores the need for dental professionals to provide training to better integrate OHE into curricula and enhance teachers' participation in oral health activities.^9^, ^15^

Additionally, this study revealed that special education teachers were disinclined to engage with parents regarding children's dental health or to ask them about the regularity of children's dental visits. Kranz et al.^15^ showed that teachers were more likely to participate in oral health activities with children than with parents. Similarly, a cross‐sectional study of 511 teachers in India illustrated that during parent–teacher meetings, 55% of teachers did not address children's oral health.^16^ Parental engagement is vital for ensuring the success and effectiveness of school‐based OHP and promoting children's adoption of healthy oral hygiene practices in school and home settings.^17^ This highlights the necessity for schools to establish clear guidelines for parental involvement, emphasising the importance of regular dental visits and discussions about oral health to extend oral hygiene support beyond the classroom.

The World Health Organization's school OHP framework offers a holistic strategy for enhancing oral health in schools, including OHE curricula, supervised toothbrushing and sugar‐restricted diets. These strategies can improve students' oral health, knowledge, behaviours and quality of life.^2^, ^18^ In our study, there was a statistically significant association between teachers' extensive involvement in OHE and the belief that students should brush their teeth at school. Only 39.0% of teachers, however, agreed with toothbrushing at school. This aligns with findings from other studies that have demonstrated low teacher participation in such activities.^16^, ^19^

Additionally, previous studies have reported challenges in implementing toothbrushing programmes, such as educator shortages, communication issues, inadequate information and resources, high staff turnover, limited parental support and the extra burden on staff assuming pseudo‐parental roles.^19^, ^20^ Despite these challenges, supervised toothbrushing programmes are cost‐effective and reduce tooth decay and health disparities in children, and some have demonstrated successful outcomes and implementation.^19^ A Saudi Arabian study showed that a toothbrushing programme significantly improved oral health for SWDs and significantly reduced dental plaque.^6^ Another study investigated the effectiveness of a Kuwaiti toothbrushing programme for children and young adults with Down syndrome and showed that it resulted in significantly improved oral health and reduced dental plaque and gingivitis.^7^ Policymakers should consider implementing supervised toothbrushing to enhance dental hygiene practices for SWDs, providing necessary resources and guidelines for implementation.

Most participants stated that school food should be sugar‐free. Similarly, a survey conducted in Sapucaia do Sul, Brazil, with 288 teachers, found that most teachers supported healthy eating and restricting low‐nutrient cafeteria options and opposed the sale of high‐sugar foods at school.^21^ The results indicated that most teachers had positive attitudes towards incorporating OHE into curricula. Similarly, a recent cross‐sectional study involving 252 schoolteachers in the Najran region of Saudi Arabia indicated that most favoured including OHE in the school curriculum.^22^ Despite this generally positive attitude towards implementing a sugar‐free policy and integrating OHE into the school curriculum, as observed in our study, further research is vital to evaluate the overall understanding of these policies among teachers and students, their actual implementation in schools and the impact on SWDs.

The participants identified multiple barriers to OHE, with 62.5% facing difficulty in finding dentists to treat SWDs. A systematic review that assessed caregivers' perspectives of the barriers faced by SWDs in using dental services emphasised the unwillingness of dentists to treat SWDs.^23^ To address this, a list of dentists who accommodate SWDs should be provided by local health authorities, and collaborative efforts should be made for healthcare professionals to train teachers to recognise and promptly address oral health issues.^9^ Additionally, policymakers might consider establishing accessible on‐site clinics or mobile dental units that travel to schools. Most subjects indicated insufficient teaching and oral health resources as barriers to implementing OHE within schools.^9^, ^14^ Collaborating with dental schools can help develop age‐appropriate, disability‐specific educational materials, fostering experience sharing and effective resource allocation for diverse student needs.

This study showed significant barriers to teachers' school‐based OHE involvement, including the misconception among 37.1% of highly involved teachers that painful primary teeth should be removed. The importance of treating primary teeth is often underappreciated due to prevailing beliefs that they are not as important as permanent teeth.^24^, ^25^ Hence, it is necessary to target education to address caregivers' common misconceptions about the importance of protecting primary teeth.^25^ There was a statistically significant association between teachers' limited participation in OHE (44.2%) and their perceptions of insufficient information about OHE (*p* = .001). Similarly, a study conducted in Greece found that limitations to implementing preschool OHE primarily resulted from teachers' inadequate self‐confidence in oral health knowledge.^26^ Most teachers highly involved in OHE believed that students should brush their teeth in school, with the results showing a statistically significant relationship. Similarly, Liontou et al.^26^ proved that teachers who participated in OHE programmes mostly reported incorporating toothbrushing into their school routines.

Teachers' demographics did not have a statistically significant effect on their perceptions of SWDs' difficulties with accessing oral health care. Similarly, a study of caregivers found that demographic factors (e.g., age, gender and education level) had no significant impact on reported difficulties for SWDs accessing dental care.^27^ The most frequently cited barriers to SWDs accessing oral healthcare services were lengthy waiting lists and times and fear of the dental clinic environment. Similarly, a retrospective cross‐sectional study examining 1611 patient records between 2009 and 2015 at the University Dental Hospital in Dammam, Saudi Arabia, showed that major barriers to seeking dental care were extended waiting periods, fear of dental treatment and difficulties securing appointments.^28^

The low prioritisation of dental services for SWDs was identified as a notable barrier to access to dental health care. Similarly, studies in Australia and Brazil reported low prioritisation within the dental care system for individuals with disabilities as a barrier to accessing public dental services.^29^, ^30^ Many respondents identified dentists' lack of training in treating SWDs as a noticeable barrier to SWDs' access to dental health care. This aligns with previous research in diverse global regions with differing healthcare systems, which consistently reports deficiencies in dentists' training as a common barrier for people with disabilities.^31^ According to the teachers, crucial barriers limiting these students' access to proper care are dental treatment costs and inadequate physical access to dental facilities, including poor clinic design, insufficient parking for disabled students and mobility difficulties. Similar findings were reported by a study in Qatif, Saudi Arabia, which examined barriers from the perspective of caregivers and revealed that physical accessibility and affordability were among the most frequently reported challenges to accessing dental health care.^27^

The generalisability of our results relies on the diverse participation of teachers from special education programmes in Saudi Arabia's Al‐Ahsa governorate. By engaging a wide range of educators from various settings, we aimed to capture the nuances and complexities inherent in OHE implementation. This broad participation enhances the robustness and applicability of the findings, providing insights that may resonate within similar educational contexts globally. It is, however, essential to recognise the influence of local factors and variations when considering broader generalisability. Although this study provides valuable insights, we must acknowledge certain limitations. First, the findings may lack generalisability to other geographic areas. Second, the reliance on self‐reported data introduces the possibility of bias. Finally, the cross‐sectional design of the study limited our ability to establish causal relationships between variables.

This article offers insights for policymakers, educators and health professionals to improve oral health care for SWDs in Saudi Arabia's Al‐Ahsa governorate and other regions. The results may support targeted preventive treatments and enhance educational strategies to ensure SWDs' oral health.

## AUTHOR CONTRIBUTIONS

F.Y.I.A. and E.K. were responsible for designing the questionnaire. F.Y.I.A. also conducted the data collection and was primarily responsible for drafting the initial manuscript. All the authors actively participated in the data analysis and interpretation. M.T. and E.K. were particularly instrumental in making critical revisions to the draft manuscript and enhancing its intellectual content. The final version was reviewed by and received unanimous approval from all the authors.

## FUNDING INFORMATION

The authors extend their appreciation to the King Salman Center for Disability Research for funding this work through Research Group no KSRG‐2023‐025.

## CONFLICT OF INTEREST STATEMENT

The authors declare that there are no conflicts of interest.

## ETHICS STATEMENT

As written in the Methods section. The invitation letter and the link to the questionnaire were sent to the Planning and Development Department of the General Administration of Education in Al‐Ahsa, who then sent invitation emails to the Department of Special Education and school principals, which were forwarded to special education teachers. Each school independently identified eligible teachers and forwarded the survey via school email addresses. The emails contained comprehensive study details, invitations to participate and links to the anonymous online questionnaire. Before completing the questionnaire, participants provided informed consent online. Ethics approval was obtained for this study from the Human Research Ethics Committee, the University of Western Australia (file reference 2022/ET000328).

## Data Availability

The data that support the findings of this study are available from the corresponding author upon reasonable request.
